# On the Use of Response Chunking as a Tool to Investigate Strategies

**DOI:** 10.3389/fpsyg.2015.01942

**Published:** 2016-01-19

**Authors:** Christopher L. Blume, Alexander P. Boone, Nelson Cowan

**Affiliations:** ^1^Department of Psychological Sciences, University of Missouri, ColumbiaMO, USA; ^2^Department of Psychological and Brain Sciences, University of California, Santa Barbara, Santa BarbaraCA, USA

**Keywords:** chunking, switch costs, focus of attention, working memory, updating

## Abstract

In this perspective we suggest that chunking can be used as an investigative tool to determine the characteristics of other cognitive phenomena. We present an example of the usefulness of chunking multiple *responses* to aid in understanding object switch costs. Switch costs refer to the shorter response times for manipulation of the same item on two trials in a row compared to a switch between items. It is presently unclear if this result is due to structural or strategic processes. We provide a short review of past literature on switch costs and a proof-of-concept example that chunking may shed new light on this topic. We examined this question with boxes filled with numbers to be arithmetically updated and memorized. A situation in which there were two response items to be manipulated per trial eliminated or reversed the switch cost effect. We suggest that participants use a strategy in which the two output responses were chunked together, making it unfeasible to prepare separately for a repetition of the most recent item as participants do in other circumstances. Our data suggest that even a well-studied phenomenon can benefit theoretically from the use of chunking as a research tool.

## Introduction

The concept of mentally combining several stimuli to form a single, meaningful unit or chunk in working memory has existed over half a century (Miller, 1956). Although the original purpose of discussing chunking was to understand how to assess the capacity of working memory, we detect that chunks can be elicited to serve another experimental purpose. Chunking can be used as an investigative tool to examine processing strategies. One can distinguish between mental *structures*, which would presumably process information in the same way, automatically, no matter what the participant’s intentions, and *strategies*, which are at least partly under volitional control of the participant. For Miller, strategies were involved in the process of converting items to chunks, as when a binary numerical representation is converted to decimal, to decrease greatly the number of chunks to be retained in working memory (e.g., 100101 = 37).

We term the kind of chunking that Miller (1956) investigated *input chunking* but we also can point to two other types. In *task chunking*, the participant is encouraged to learn a sequence of tasks that reliably repeat. For example, Koch et al. (2006) required reporting of the form, size, or color of an item and found an advantage for a known sequence of these tasks, but only when the participant was made aware of the repeating sequence. This is not a foregone conclusion, because task chunking was found to be strategic when it could have been structural (present regardless of the participants’ awareness).

Here we point out a third type of chunking that we term *response chunking*. This phenomenon can occur when the task requirements are such that two or more stimuli are to be followed by multiple responses. The participant has the choice of carrying out some requisite processing for Stimulus *A* and then responding to *A*, followed by processing *B* and then responding to *B*; or, alternatively, processing both *A* and *B* and then responding to both in turn. As we will see in our empirical example, this strategic difference is important and can shed light on whether the underlying processing is itself open to strategic influences.

A particularly robust effect in working memory research for which we illustrate the strategic basis, using response chunking, is that of object switch costs. Switch costs refer to the additional time it takes to respond to Trial *n* compared to Trial *n-1* when Trial *n* is a response to a new item as opposed to a repetition of the same item as in Trial *n-1*. Switch costs are large magnitude effects (e.g., Oberauer, 2002), which might be taken as evidence that they are structural and automatic rather than strategic in origin. In fact, though, whether switch costs are the result of some inevitable, structural process, such as the distinctiveness of a memory trace from the most recently completed cognitive process, is a fundamental issue yet to be resolved. There is at least one example of switch cost effects that appear dependent on specific task demands. Gehring et al. (2003) examined the speed of incrementing counts for two streams of symbols, with “#” and “@” assigned to one stream and “&” and “%” assigned to the other stream. In some sequences of trials, a switch from one stream to the other was unexpectedly advantageous compared to no switch, possibly because of expectations (e.g., the expectation of a switch following the sequence *12121_*). Rather than replicate Gehring et al. (2003) we chose to focus on the potential effects of chunking with regard to potential variability of switch costs.

There are few examples of chunking within the switch cost literature, and what examples there are do not reference the kind of chunking we wish to test. Oberauer and Bialkova (2009) and Gilchrist and Cowan (2011) examined the effect of input chunking: they required participants to respond to stimuli based on the combination of two features. The conclusion of Oberauer and Bialkova (2009) was that the focus of attention was limited to a single chunk no matter whether that chunk was a single item, or a learned pairs (two color-number associations). Gilchrist and Cowan (2011), on the other hand, found a situation in which two associations could be held in working memory either separately (e.g., triangle-z and red-2) or as a single chunk (e.g., with the association between triangle-z and red-2 learned). When the associations were held separately, changing either one of them between trials slowed response times (RTs), but changing both of them slowed RTs even more. After the two associations were combined into a learned chunk, changing either of them slowed RTs just as much as changing both of them. Thus, after an input chunk is formed, repetition is beneficial but changing part of the chunk requires that the individual encode the entire set anew (cf. Oberauer and Bialkova, 2011). These papers were directed at understanding the fundamental limit of the focus of attention, not the role of strategies *per se*. It would be possible to extend this research to examine strategies, for example by showing that a learned association can be either used or ignored, with different outcomes.

We examine response chunking, and use it to ask whether switch costs themselves are the outcome of a strategic configuration in the mind rather than being automatic or structural. After Oberauer (2002), we used sets of three boxes with a digit in each box. Whereas Oberauer (2002) proceeded to ask participants to update a single box per trial (e.g., +3), we required that *two* boxes be updated on each trial. This arrangement allowed six different sequences relating Trial *n-1* to Trial *n*, as shown in **Table 1** (left-hand column). The same two boxes could be updated in the same order on these two successive trials (denoted *AB/AB*), the order of updating could switch (*AB/BA*), and/or the previously non-updated box could be updated (e.g., *AB/CA*). The expectations pertain to the six trial types shown in the table.

**Table 1:** Response times as a function of condition.

Condition	Response 1 (*SEM*)	Response 2 (*SEM*)	Trials possible	Trials completed
*AB/AB*	3575 (171)	2319 (168)	864	630
*AB/AC*	3548 (160)	2494 (173)	288	201
***AB/A***	**3561 (159)**			
*AB/BA*	3486 (211)	2391 (149)	864	639
*AB/BC*	3366 (202)	2530 (210)	288	203
***AB/B***	**3426 (200)**			
*AB/CA*	3407 (244)	2584 (184)	288	219
*AB/CB*	3245 (173)	2277 (187)	288	199
***AB/C***	**3326 (195)**	-	-	-

*Completed trials refer to correct trials; the trial block ended after the first mistake. Condition can be read with each letter representing a random box. For example, Condition AB/CA represents a Trial in which any Box A and Box B are updated in turn, followed by a trial in which Box C and then A are updated. The letters do not reflect ordinal positions; e.g., on some trials A is the middle box, B is the right box, and C is the left box. Mean response times are in milliseconds, with standard errors of the mean in parentheses.*

We suspect that individuals often seek the least effortful means to complete the task, which should depend on the exact setup. With the single-switch procedure, the least effortful means is to retain the task set on Trial *n-1* in case it is still relevant on Trial *n*. By making the situation more complex, we open up the possibility of a change in what is strategically best. It might be strategically best to preserve the last response without any chunking, but that response would only be preserved on 1/3 of the trials (third and fourth rows of **Table 1**). If that is what is found, the results will be the same as in previous studies so we will not be able to rule out the possibility that switch costs are indeed automatic. However, it alternatively might be strategically best to encode a pair of responses as a chunk that can be preserved for the next trial, though that same pair would only repeat on 1/6 of the trials (first row of the table). If neither of these strategies is fruitful, the participant could throw out each pair and start anew. If this strategy is taken, there could be a switch benefit rather than a switch cost, as interference from repetition is avoided when there is a switch. Notice that the last two strategies would produce results different from the usual switch cost, and therefore would demonstrate that switch costs are strategic, not automatic.

## Methods

### Participants

Summer undergraduate psychology course credit was awarded to 24 participants (14 female, 10 male; mean age 21.21 years, *SD* = 1.67). Four additional participants were excluded; two because of a native language other than English and two because they did not make any correct responses in at least one condition. This study was carried out in accordance with the ethical guidelines of the American Psychological Association and was approved by the University of Missouri Institutional Review Board. All subjects gave written informed consent in accordance with the Declaration of Helsinki.

### Apparatus, Stimuli, and Procedure

With stimulus sizes based on Oberauer (2002), participants viewed three boxes on the screen, each with a single digit inside. The participant memorized the digits and then pressed the SPACE key to continue. Differences from Oberauer were (1) the use of only one row of boxes per trial, and (2) the requirement of two concurrent operations at each step as opposed to only one. In particular, two of the three digits were replaced by arithmetic operations (e.g., *+2* and *-3*) such that each result remained 1–9. Each trial had one arithmetic update highlighted with a green box, signifying this should be the first response provided, and another in a black box, for which the second response on the trial was to be given. (The green box was a different box from trial to trial and the response order was random, rather than always from left to right.) Participants were to solve the equation and key in responses as quickly and accurately as possible, using the top row of number keys on a standard QWERTY keyboard. The response digit was also to replace the previous digit in the target box for subsequent trials in the block.

The experiment thus posed a strategic choice as to whether the responses should be converted to a single chunk or maintained uniquely from each other. Given that there were three boxes, at least one of the boxes was updated on both Trial *n-1* and Trial *n*.

There were six key conditions that could take place in the experiment. (1) An *AB/AB* condition is a repetition of the same two targeted items, *A* and *B*, requiring responses in the same order on two consecutive trials. If the response is converted to a two-item chunk, repetition of the chunk leads to the prediction of the shortest RTs of any condition. (2) An *AB/BA* condition is a repetition of the most recent element and should provoke the shortest first response if there is no chunking and each item is considered individually. (3) An *AB/BC* condition contains the same repetition as Condition 2 and should have as short of a first RT if there is no chunking. (4) An *AB/AC* condition contains a non-immediate repetition for the first response, like Condition 1. (5) An *AB/CA* condition contains a non-immediate repetition for the second response. (6) An *AB/CB* condition contains a non-immediate repetition for the second response.

There were eight practice blocks and 24 experimental blocks of six trials (12 arithmetic updates) per block. Trials 2, 4, and 6 in a block always included the same two boxes as Trial *n-1*, though the response order could be reversed, whereas Trials 3 and 5 always included the box *not* selected on Trial *n-1*. Data were excluded from analyses following the first incorrect response in a trial, regardless of recovery from an error later in a trial sequence. Individual trial RTs less than 0.5 s were excluded because they were thought to represent primarily guesses, and those longer than 10 s were excluded because they were thought to represent inattention to the task. These exclusion parameters resulted in approximately 27% of all trials from analyses, almost all from errors during a trial block.

## Results

**Table 1** shows the mean RT for the first and second responses in each of the six key conditions. RT1 is more theoretically diagnostic because RT2 is dependent on processing related to RT1. If switch costs were automatic and applied at the individual-response level, then one would expect a cost when the first response of Trial *n* occurred on a different box than the second response of Trial *n-1*, compared to when they occurred on the same box (e.g., a faster RT1 of Trial *n* for *AB/BA* than for *AB/CA*.). If switch costs were automatic but applied to the pair of responses together, then one would expect a cost when the first response of Trial *n* occurred on a different box than the *first* response of Trial *n-1* (e.g., a faster RT1 of Trial *n* for *AB/AB* than for *AB/BA*). As one can see, neither of these structural predictions were supported in the data.

We conducted a Bayesian ANOVA (Rouder et al., 2009) of all of the data with two within-participant factors: response serial position (1 or 2) and task condition (1–6 corresponding to the rows of **Table 1**). The ANOVA shows the best model was one that took into account only the response position, Bayes Factor (*BF*) = 15.61e + 31. This model accounts for 94.10% of the error. Condition, in contrast, could not be said to lead to a difference in RT, *BF* = 0.017, or 59 to 1 in favor of a null effect.

Our subsequent analyses were designed to get a more powerful view of possible effects of switches on RT1, which was of most theoretical interest given that RT2 may be contaminated by RT1 processing. We carried out a second Bayesian ANOVA on RT1 alone (*BF* = 0.254) and Bayesian *t*-tests on RT1 separately for the six conditions in the experiment. There was no switch cost on RT1 (**Table 1**). In fact, we found some switch benefits. All paired-sample, two-tailed tests provided anecdotal-to-moderate evidence for no difference between conditions (Rouder et al., 2009), BF between 0.222 and 0.707, with two exceptions. The RT for the pure chunk replication condition, *AB/AB* (*M* = 3575 ms, *SEM* = 171 ms), was *longer* than *AB/CB* (*M* = 3245 ms, *SEM* = 173 ms), *BF* = 13.61. This provides strong evidence that these two conditions differ in RT. Likewise, the *AB/AC* (*M* = 3548 ms, *SEM* = 160 ms) condition was longer than *AB/CB, BF* = 4.76. This provides moderate evidence that these two conditions differ in RT. In each case the difference in RT was opposite the direction of a predicted switch cost. Close inspection of the table and the analyses show a trend suggesting that whenever *C* is included, as the first or second response, then RT1 benefits. This pattern can be attributed to the notion that the inclusion of *C* signals to the participant that the previous chunk should be discarded.

Nevertheless, when one collapses across the second response for simplicity (see **Table 1**, bolded rows for means), the basic trends hold: there is no overall effect in the Bayesian ANOVA, *BF* = 0.63; in *t*-tests, *AB/A* = *AB/B, BF* = 0.37; *AB/A* > *AB/C, BF* = 2.98; and *AB/B* = *AB/C, BF* = 0.31.

## Discussion

In our experiment we were able to completely negate the switch cost effect by requiring multiple responses on a single trial. Furthermore, we actually *reversed* the direction of the effect so that participants showed some switch benefits. Participants were able to respond the fastest when the least amount of information was carried over from Trial *n-1* to Trial *n*, in direct contrast to the switch cost effect when only one item is updated per trial. The greatest RT disparity occurs between the *AB/AB* and *AB/CB* conditions. The former condition simply repeats a chunk the same way a single update version of the task (e.g., Oberauer, 2002) repeats a single item. Despite the similar conditional parameters, the dual-update trials now result in the longest RT rather than the shortest. The *AB/CB* condition requires that the one item not in mind on the previous trial be brought to mind; this results in the shortest RT. These data seem to indicate information in mind from a previous trial will interfere with the ability to respond on following trials. The difficulty appears to be in disconnecting each item from a two-item chunk from the previous trial in order to make the response. This interpretation is in line with past literature on response inhibition (Koch et al., 2010), interference (Carroll et al., 2010), and updating sets of items (Kessler and Meiran, 2006).

In this short experiment we hoped to illuminate a portion of the as yet unidentified nature of the switch cost effect by introducing a chunking strategy. The present results are in line with the assertion that, when requiring two responses per trial, a response chunking strategy can negate the switch cost effect. Participants responded either as fast or faster when a condition introduced a new item on a particular trial. An updating task with only one item in mind appears conducive to the strategy of preparing for the same item repeatedly (or at least failing to disengage attention from that item). However, an updating task that requires at least two items in mind does not appear to engender the same strategy. It may be that a chunking strategy is used rather than a switching strategy.

Participants were able to provide the second response per trial in each condition about 1s faster than the first responses. However, the RT in each case is over 2 s, which seems to be longer than should be necessary to simply find and press a key. We account for this relatively long RT as another result of the participant’s strategy during the task. After providing RT1, the only time available to prepare for the upcoming trial was between RT1 and RT2, so we believe that participants prepared for Trial *n+1* during this inter-response interval. Similarly, Kessler and Meiran (2006) showed that the set size of non-updated items can increase the RT costs associated with the procedure. In other words, when an update occurs to one item from a set of items, the entire set must be updated. We posit from the current data that the full set update occurs following Response 1, but prior to Response 2.

## Conclusion

In our experiment, we were able to reverse the switch cost effect by setting up a situation demanding strategies that discouraged retention of the pre-switch objects. No switch cost was ever produced even when compared to trials on which the same single item was updated consecutively (*AB/BA*). RT appears to show that participants attempted to clear the contents in mind from the previous trial to avoid interference between Trial *n-1* and Trial *n*.

This research represents an initial foray into the determination of whether switch cost effects are the result of some automatic or strategy-based process. Our results support a strategy-based interpretation of switch costs when multiple items can be chunked together within a trial, but more research is needed to sufficiently resolve this issue. The updating procedure and resulting switch costs can be a promising avenue of work on how, or if, individuals are able to chunk multiple output responses. From the literature and our own data, we believe that it is possible to distinguish between three ways in which chunking processes can be used as a tool that illustrates the strategic nature of processing, which we have termed input, task, and response chunking.

We have focused on the usefulness of response chunking to understand the strategic nature of switch costs, which previously might have seemed like automatic effects. A similar research strategy could be useful to examine other effects that seem ubiquitous, e.g., various stimulus-response compatibility effects and Stroop-like effects. For example, if trials are presented in pairs and most of the time a congruent response follows an incongruent one, then the rare presentation of a congruent–congruent trial pair should violate expectations for the second trial and rupture the strategy that has developed across the task, slowing the second-trial RT and thus revealing the strategic nature of the task.

## Conflict of Interest Statement

The authors declare that the research was conducted in the absence of any commercial or financial relationships that could be construed as a potential conflict of interest.

The reviewer and handling Editor declared their shared affiliation, and the handling Editor states that the process nevertheless met the standards of a fair and objective review. Neither the reviewers nor the handling Editor share an affiliation with the authors.
